# High prevalence and incidence of sexually transmitted infections among women living in Kwazulu-Natal, South Africa

**DOI:** 10.1186/1742-6405-11-31

**Published:** 2014-09-15

**Authors:** Sarita Naidoo, Handan Wand, Nathlee Samantha Abbai, Gita Ramjee

**Affiliations:** 1 HIV Prevention Research Unit, South African Medical Research Council, Durban, South Africa; 2 National Center for HIV Epidemiology and Clinical Research, Sydney, Australia; 3 Department of Epidemiology and Population Health, London School of Hygiene & Tropical Medicine, London, UK

**Keywords:** Sexually transmitted infections, HIV, Kwa-Zulu Natal, Women, Prevalence, Incidence, Risk factors

## Abstract

**Background and objectives:**

Sexually transmitted infections (STIs) contribute largely to the burden of health in South Africa and are recognized as major contributors to the human immunodeficiency virus (HIV) epidemic. Young women are particularly vulnerable to STIs. The purpose of this secondary analysis was to examine the risk factors associated with prevalent and incident STIs among women who had participated in three clinical trials.

**Methods:**

A total of 5,748 women were screened and 2293 sexually active, HIV negative, non-pregnant women were enrolled in three clinical trials in Kwazulu-Natal, South Africa. The prevalence of individual STIs *Chlamydia trachomatis* (CT), *Neisseria gonorrhea* (NG), syphilis, and *Trichomonas vaginalis* (TV) was assessed at screening; and incident infections were evaluated over a 24 month period.

**Results:**

Overall, the combined study population of all three trials had a median age of 28 years (inter-quartile range (IQR):22–37), and a median duration of follow-up of 12 months. Prevalence of STIs (CT, NG, TV, or syphilis) was 13% at screening. The STI incidence was estimated to be 20/100 women years. Younger women (<25 years, p < 0.001), women who were unmarried (p < 0.001) and non-cohabiting women (p < 0.001) were shown to be at highest risk for incident STIs.

**Conclusions:**

These results confirm the extremely high prevalence and incidence of STIs among women living in rural and urban communities of KwaZulu-Natal, South Africa, where the HIV epidemic is also particularly severe. These findings strongly suggest an urgent need to allocate resources for STI and HIV prevention that mainly target younger women.

**Trial registration:**

Clinical Trials.gov, NCT00121459.

## Introduction

South Africa (SA) is at the epicenter of the human immunodeficiency virus (HIV) epidemic; and other sexually transmitted infections (STIs) continue to be endemic [1-3]. STIs have been associated with pelvic inflammatory disease, adverse pregnancy outcomes, cervical cancer, infertility, and multiple reproductive tract sequelae [4]. Furthermore, the evidence that STIs facilitate HIV acquisition and transmission [5,6] enhances the seriousness of the problem.

KwaZulu-Natal (KZN), the most densely populated province in SA, has been markedly affected by both the HIV and STI epidemic [7,1]; with a disproportionate burden of STIs and HIV among women. Several studies conducted within various populations of women in KZN have shown high prevalence of HIV coupled with STIs [8-11]. Control of STIs therefore remains extremely important in these high risk populations. Availability of data on the epidemiology of STIs and associated risk factors in these populations is thus essential for the development of successful targeted interventions in this region.

This study presents combined data from a large cohort of women, from urban and rural areas of KZN, who participated in three clinical trials; to determine the prevalence and incidence of STIs [(*Chlamydia trachomatis* (CT), *Neisseria gonorrhea* (NG), syphilis, and *Trichomonas vaginalis* (TV)] and the associated risk factors. This data provides an opportunity to gain further insights into the overall STI prevalence and incidence in this region; and will inform the design of future HIV/STI prevention strategies by identifying and defining populations most at risk.

## Methods

### Study population

This analysis includes women who participated in three multi-centre trials at sites in KZN: Methods for Improving Reproductive Health in Africa (MIRA) trial [12], conducted in rural Umkomaas and Botha’s Hill; Microbicides Development Programme (MDP) Feasibility study in preparation for Phase III Microbicide Trials [13], in semi-rural Tongaat and Verulam; and HIV Prevention Trials Network (HPTN) Site Preparedness study (HPTN 055) for future implementation of Phase 2/IIb/III HIV Prevention Studies [14], in an urban area in Durban and in a rural district of Hlabisa. Study populations were described elsewhere in detail [12-14].

### Study procedures

Study procedures were described in detail elsewhere [12-14]. Main inclusion criteria were similar in all three studies, and briefly included being sexually active; HIV negative at screening; not pregnant, willing to provide written consent, and follow study procedures. Information on demographics and sexual behaviors were collected. Participants received risk reduction counseling and condoms; and treatment was provided for curable, symptomatic or laboratory-diagnosed STIs. Women diagnosed as HIV-positive were referred to local health care facilities for further care and support. Male partners were encouraged to access the trial sites for counseling, HIV testing and STI treatment. Additionally, women diagnosed with STIs were given referrals for their partners to access treatment at local clinics. Study protocols and informed consent forms were approved by the local ethics committee and various Institutional Review Boards.

### Laboratory procedures

STI and HIV testing was described previously for each study [12,15,16]. Briefly, in the MIRA trial, [16] CT, GC and TV were detected by PCR (Roche Pharmaceuticals, Branchburg, NJ, USA) and syphilis by rapid plasma reagin (RPR) and Treponema pallidum haemagglutinin (TPHA) (Randox Laboratories, Crumlin, UK). In HPTN 055 [15], CT and GC were detected using the BDProbe Tec ET assay (Becton Dickinson, MD), TV by wet mount microscopy and syphilis by RPR and confirmatory TPHA. In MDP Feasibility [16], CT and GC were detected using PCR (COBAS Amplicor, Roche Molecular Diagnostics, Pleasanton, CA, USA), TV by wet mount microscopy and syphilis by RPR and confirmatory TPHA (Omega Diagnostics, Alva, UK).

### Statistical analysis

Differences in proportions were tested using the chi-square test. The Student’s t test was used to compare averages. Univariate and multivariate logistic regression analyses were conducted to identify variables predictive of any STI (CT, NG, TV or Syphilis) infection at screening. Only variables determined to be consistent across the studies were included.

STI incidence was determined as the first positive test after a negative test at enrollment. Associations between various factors and STI incidence were described using Kaplan-Meier survival plots and log-rank tests. Risk factors for STI incidence were assessed using Cox proportional hazard regression models. Multivariate models considered all variables statistically significant (p < 0.05) in initial analyses and used forward stepwise methods. Statistical analysis was performed using STATA Release 8.2 (Stata Statistical Software: Stata Corporation, College Station, Texas, USA).

## Results

Of the 5,748 women screened, 3492 were from the MIRA trial, 1221 from MDP Feasibility; and 1035 from HPTN055. Overall, the women had a median age of 28 years (inter-quartile range (IQR):22–37), and a median duration of follow-up of 12 months (data not shown). Majority were from rural areas (91%); unmarried (85%); and not living with regular sexual partners (non-cohabiting) (68%). Over half (53%) reported less than high school education. HIV and STI (diagnosed with at least one STI: CT, NG, TV or syphilis) prevalence at baseline was 42% (2407/5748) and 13% (738/5748), respectively.

### Risk factors for STIs at screening

Women with prevalent STIs (≥1) were younger (<25 years) (p < 0.001); reported less than high school education (p < 0.0001), were unmarried (p < 0.0001); and non-cohabiting (p < 0.0001) (Table 1). Table 2 shows that women who were HIV positive, <25 years of age; unmarried; non- cohabiting; and who reported less than high school education were significantly associated with increased risk of having a prevalent STI (Odds Ratios (OR):1.50, 95% Confidence Intervals (CI):1.27-1.76; OR:1.36, 95% CI:1.11-1.68; OR:1.60, 95% CI:1.14-2.24; OR:1.27,95% CI:1.03-1.60; and OR:1.75, 95% CI:1.49-2.06 respectively).

**Table 1:** **Demographic variables by STI**^**† **^**status at screening**

**Variables**	**Overall**	**STI+**	**STI-**	**p-value**
	**N = 5,748 (%)**	**N = 728 (%)**	**N = 5,020 (%)**	
**HIV prevalence at screening**	2,407 (42)	375 (52)	2,032 (40)	<0.001
**District**				0.524
Rural^‡^	5216 (91)	656 (90)	4560 (91)	
Urban^ᆕ^	532 (9)	72 (10)	460 (9)	
**Age, mean (SD)**	28.6 (8)	26 (7)	29 (8)	
**Age groups (years)**				
≤ 24	2,223 (39)	367 (50)	1856 (37)	<0.001
25-34	2,224 (39)	255 (35)	1969 (39)	
35+	1,305 (22)	106 (15)	1199 (24)	
**Education**				
Less than high school	3069 (53)	467 (61)	2602 (52)	<0.0001
**Marital status**				
Not married	4,912 (85)	677 (93)	4235 (84)	<0.0001
**Co-habitation status**				
Non-cohabiting	3,929 (68)	566 (78)	3363 (70)	<0.0001

^†^Diagnosis of chlamydia, gonorrhea, trichomonas or syphilis.

^‡^Hlabisa, Umkomaas, Botha’s Hill, Tongaat and Verulam.

^ᆕ^Durban.

**Table 2:** Risk factors for being diagnosed with STI at screening: results from univariate and multivariate logistic regression models

**Variable**	**Univariate analysis**	**p-value**	**Multivariate analysis**	**p-value**
	**OR (95% CI)**		**OR (95% CI)**	
**HIV infection status**				
HIV negative	1		1	
HIV positive	1.56 (1.34-1.83)	<0.0001	1.50 (1.27-1.76)	<0.0001
**Age (years)**				
<25	1.60 (1.33-1.92)	0.000	1.36 (1.11-1.68)	0.003
25 - 34	1.17 (0.95-1.43)	0.137	1.08 (0.87-1.34)	0.480
35+	1	-	1	-
**Education**				
Less than high school	1.67 (1.42-1.96)	<0.00001	1.75 (1.49-2.06)	<0.0001
**Marital status**				
Not married	2.47 (1.84-3.32)	<0.0001	1.60 (1.14-2.24)	0.006
**Co-habitation status**				
Non-cohabiting	1.73 (1.43-2.07)	<0.0001	1.27 (1.03-1.60)	0.024

CI, confidence interval; OR, odds ratio.

### Incidence of STIs

**Figure 1:**
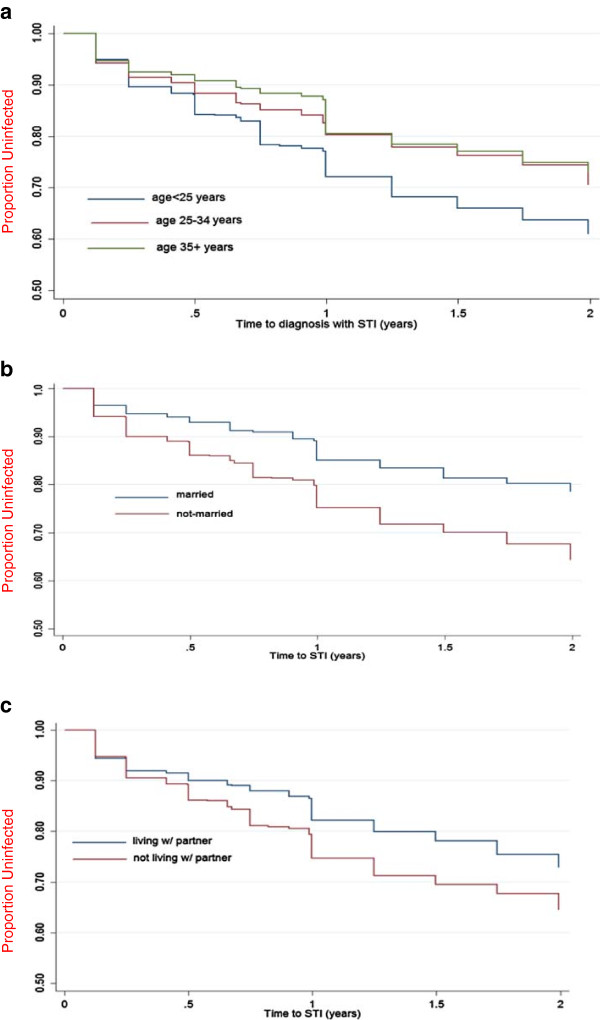
The Kaplan-Meier survival curves of STI incidence stratified by age (a), marital status (b) and cohabitation status (c).

Crude STI incidence amongst the 2293 enrolled women was 680 (20/100 women-years). Kaplan-Meier survival curves of infection stratified by age, marital, and co-habitation status are shown in Figure 1a-c. The highest incidence rate was observed for women <25 years old (26/100 women-years, p < 0.001), followed by moderately high incidence rates for unmarried and non-cohabiting women (22/100 women-years, p < 0.001 and 21/100 women-years, p < 0.001; respectively).

Table 3 shows that baseline STI infection, being <25 years of age; unmarried; and non-use of contraception were significantly associated with increased risk of acquiring incident STIs (OR:1.52, 95% CI:1.22-1.88; OR:1.40, 95% CI:1.14-1.74; OR:1.54, 95% CI:1.22-1.94 and OR:1.40, 95% CI:1.19-1.64, respectively). No significant associations with incident STIs were observed for geographical area, employment status, condom use and education level.

**Table 3:** Risk factors for incidence of STI during follow up: results from cox regression models

**Variable**	**Univariate analysis**	**p-value**	**Multivariate analysis**	**p-value**
	**HR (95% CI)**		**HR (95% CI)**	
Any STI at baseline	1.61 (1.30-2.00)	-	1.52 (1.22-1.88)	0.000
**Age**				
≤25	1.64 (1.36-2.00)	<0.001	1.40 (1.14-1.74)	0.001
25-34	1.19 (0.97-1.46)	0.100	1.10 (0.89-1.37)	0.370
35+	1	-		
**District**				
Rural	0.93 (0.69-1.25)	0.628	-	
Urban	1	-		
**Employed/income**				
No	1.35 (1.10-1.66)	0.0038	-	
Yes	1	-		
**Level of education**				
High school or more	1	-		
Less than high school	1.25 (1.06-1.46)	0.006	-	
**Contraceptive use**				
At least one form of contraceptive^1^	1	-		
None	1.33 (1.13-1.55)	<0.001	1.40 (1.19-1.64)	0.000
**Marital status**				
Married	1	-		
Not married	1.84 (1.49-2.27)	<0.0001	1.54 (1.22-1.94)	0.000
**Cohabitation status**				
Yes	1	-		
No	1.43 (1.21-1.68)	<0.0001	-	
**Condom used during last sexual act**				
No	1.00 (0.86-1.16)	0.98	-	
Yes	1	-		

CI, confidence interval; HR Hazard ratio.

^1^including condom, injectables, pills, traditional methods.

## Discussion

Our analysis confirms that STI prevalence and incidence is extremely high in women living in KZN. The prevalence of STIs was 13% while the incidence rate was 20/100 women-years. Similar STI incidence was reported in other studies conducted in SA [15,17-19]. Incidence of STIs in this cohort was unacceptably high despite provision of risk reduction counseling, condoms, treatment for curable STIs; and partner treatment or referral.

In this study, women who were <25 years; unmarried; and non-cohabiting were identified as being at most risk for STI acquisition. Recent evidence from a PrEP trial, VOICE, conducted in SA, Zimbabwe and Uganda, showed the same risk factors associated with increased risk of HIV acquisition [20]. Similarly, Feldblum and colleagues [19] observed that younger age was associated with incident HIV and STIs. Another study also showed that younger women were at higher risk of acquiring new STIs [18]. Studies in Tanzania [21], Kenya [22] and Brazil [23] reported that being unmarried was associated with STI acquisition.

Predictably, having an STI at baseline also placed women at increased risk of acquiring an STI during follow up, and at substantial risk of HIV acquisition. HIV infection at screening was associated with a significantly higher prevalence among women diagnosed with at least one STI compared to those who had no STI; providing further evidence for the relationship between HIV and STIs. Mlisana and colleagues [17] reported a similar finding. It was also previously suggested that HIV prevention efforts in SA may be enhanced by screening STI patients for acute HIV infection [6].

This study defined populations who are most at risk of STI acquisition and broadly showed the common risk factors associated with STI prevalence and incidence suggesting the need for effective behavioural or biomedical interventions. This data will aide in the design of future interventions in these populations; and contributes to the STI surveillance data for women in this region.

Our study has several limitations. These results may not be representative of the wider population as these women were recruited from selected populations. However this large data set, together with the numerous STIs detected, provides valuable information on the STI epidemic in this region. Another limitation is the slight variations in testing methods used for STI diagnosis in the different trials, where some methods may have been more sensitive than others. Additionally, the risk factor analyses were restricted to socio-demographic and behavioral variables that were assessed consistently across all three studies, possibly resulting in reducing the power of the analyses to detect significant associations. Finally, although all STIs were treated, there was no information on partner infection status or resistant infections during follow up.

## Conclusions

Due to the high incidence of STIs observed in this population, targeted interventions aimed at young women and their partners, are urgently needed. Control and treatment of STIs still remains an important public health priority for HIV prevention and should include a combination of prevention efforts such as extensive health education, condom promotion, male circumcision, and HIV and STI testing in all patient-provider encounters.

## Competing interests

The authors declare that they have no competing interests.

## Authors’ contribution

GR and HW developed the concept. HW conducted the statistical analysis. SN wrote the manuscript with input from GR, HW and NSA. All authors read and approved the final manuscript.

## Authors’ information

Handan Wand, Nathlee S Abbai, Gita Ramjee Co-authors.
